# Supplementary figures and images for: Metastatic breast cancer cells induce altered microglial morphology and electrical excitability in vivo
Source: J Neuroinflammation. 2020 Mar 19;17:87. doi: 10.1186/s12974-020-01753-0 (PMC7081703; doi:10.1186/s12974-020-01753-0)

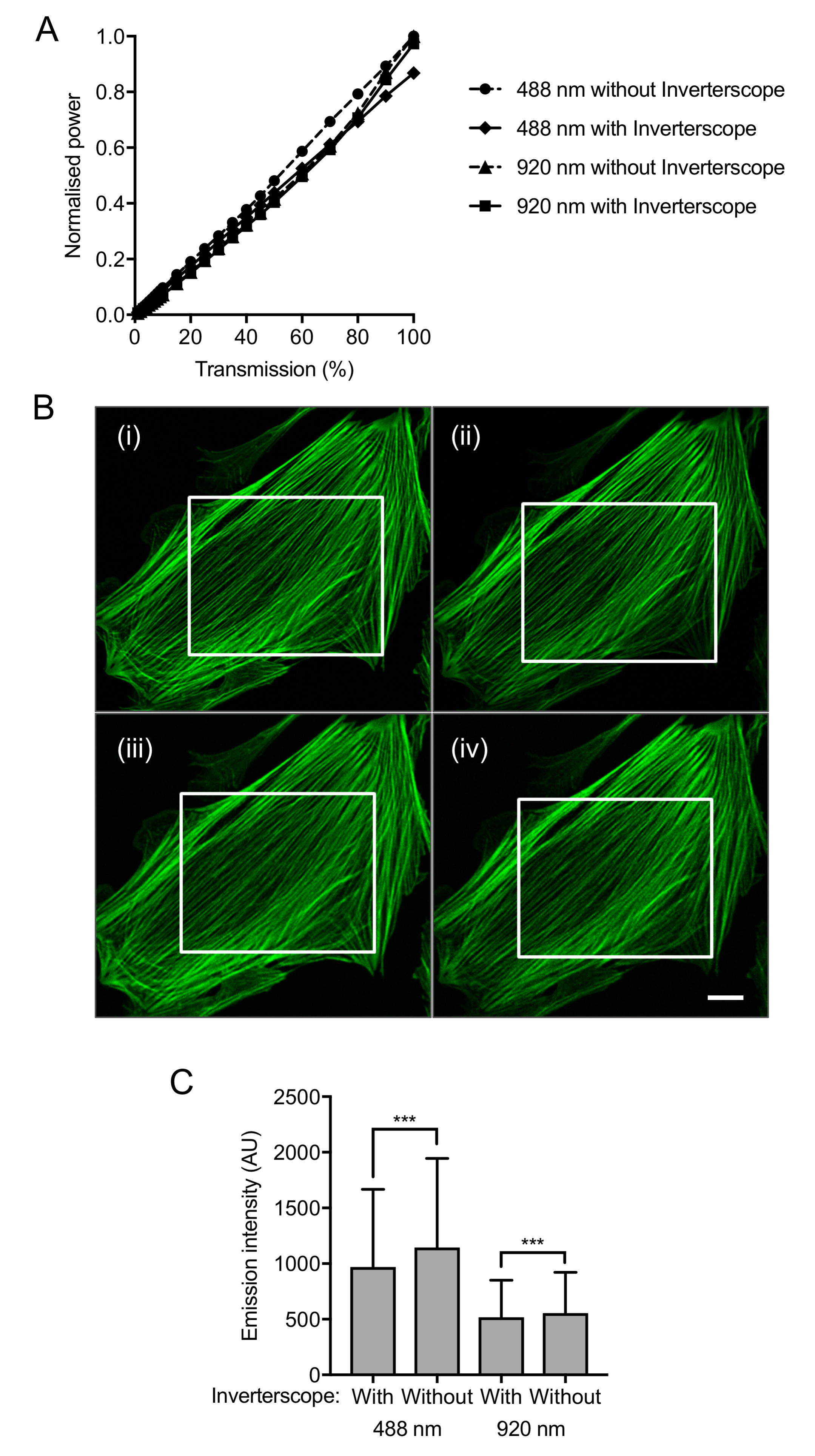

Supplement: Supplementary file 1 — Additional file 1: Supplementary Figure 1. Effect of InverterScope on laser power and emission intensity. (A) Laser power at 488 nm and 920 nm exiting the objective with and without the InverterScope, measured at the indicated transmission percentages using a Coherent Fieldmate power meter. (B) AlexaFluor 488 phalloidin emission intensity in a standard sample imaged with and without the InverterScope: (i, ii) 488 nm excitation (40 mW) with 500–550 nm emission directed to the internal detectors. (iii, iv) 920 nm 2-photon excitation (13 mW) with 500–550 nm emission directed to the non-descanned detectors. i,iii show a cell without the InverterScope and ii,iv show the same cell with the InverterScope. (C) Mean intensity measured within a defined region of interest (white box). ***P < 0.001, ANOVA with Sidak’s multiple comparisons test. Scale bar, 20 μm. Data are mean ± SD. [file 12974_2020_1753_MOESM1_ESM.tif]

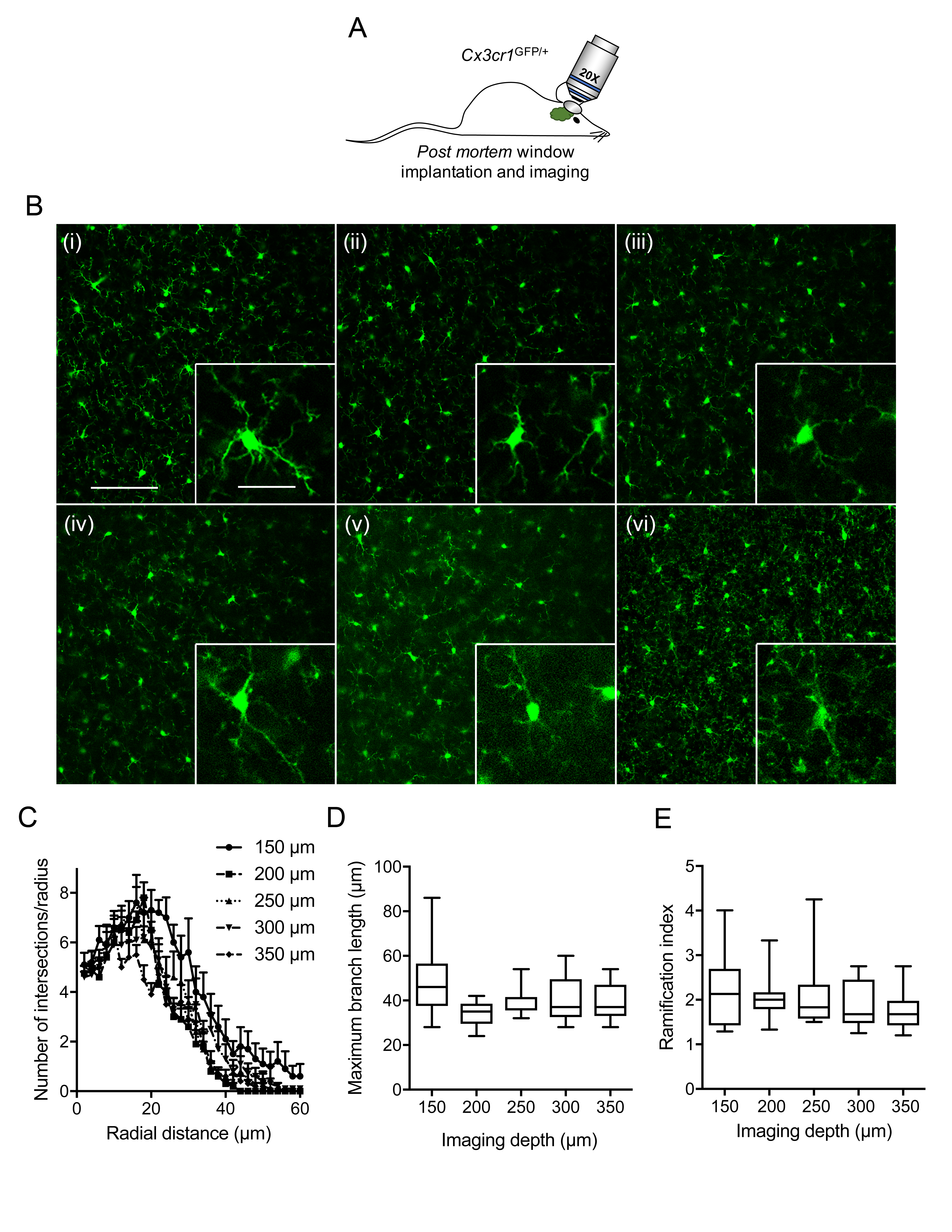

Supplement: Supplementary file 2 — Additional file 2: Supplementary Figure 2. Post mortem visualisation of GFP-expressing microglia through a cranial imaging window. (A) Cx3cr1GFP/+ mice were euthanised, imaging window implanted as described in Methods, and microglia visualised using adapted multiphoton microscope. (B) Representative images of microglia at various distances below the imaging window: 150 μm (i), 200 μm (ii), 250 μm (iii), 300 μm (iv), 350 μm (v). (vi), microglia in a coronal section of layer 2/3 cortex from a wildtype mouse labelled with anti-Iba1 antibody. Scale bar, 100 μm. Insets: 3X magnification; scale bar, 30 μm. (C) Sholl analysis plot of microglia imaged at the indicated depths below the imaging window (n = 10 cells/group). (D) Maximum branch length (μm) of microglia imaged at the indicated depths (n = 10 cells/group). (E) Schoenen ramification index of microglia at the indicated depths (n = 10 cells/group). Laser power was 6 mW for depths 150 μm – 250 μm, and 10 mW at 300 μm and 350 μm. Box plots show median, 25th and 75th percentile values; whiskers are minimum and maximum values. [file 12974_2020_1753_MOESM2_ESM.tif]

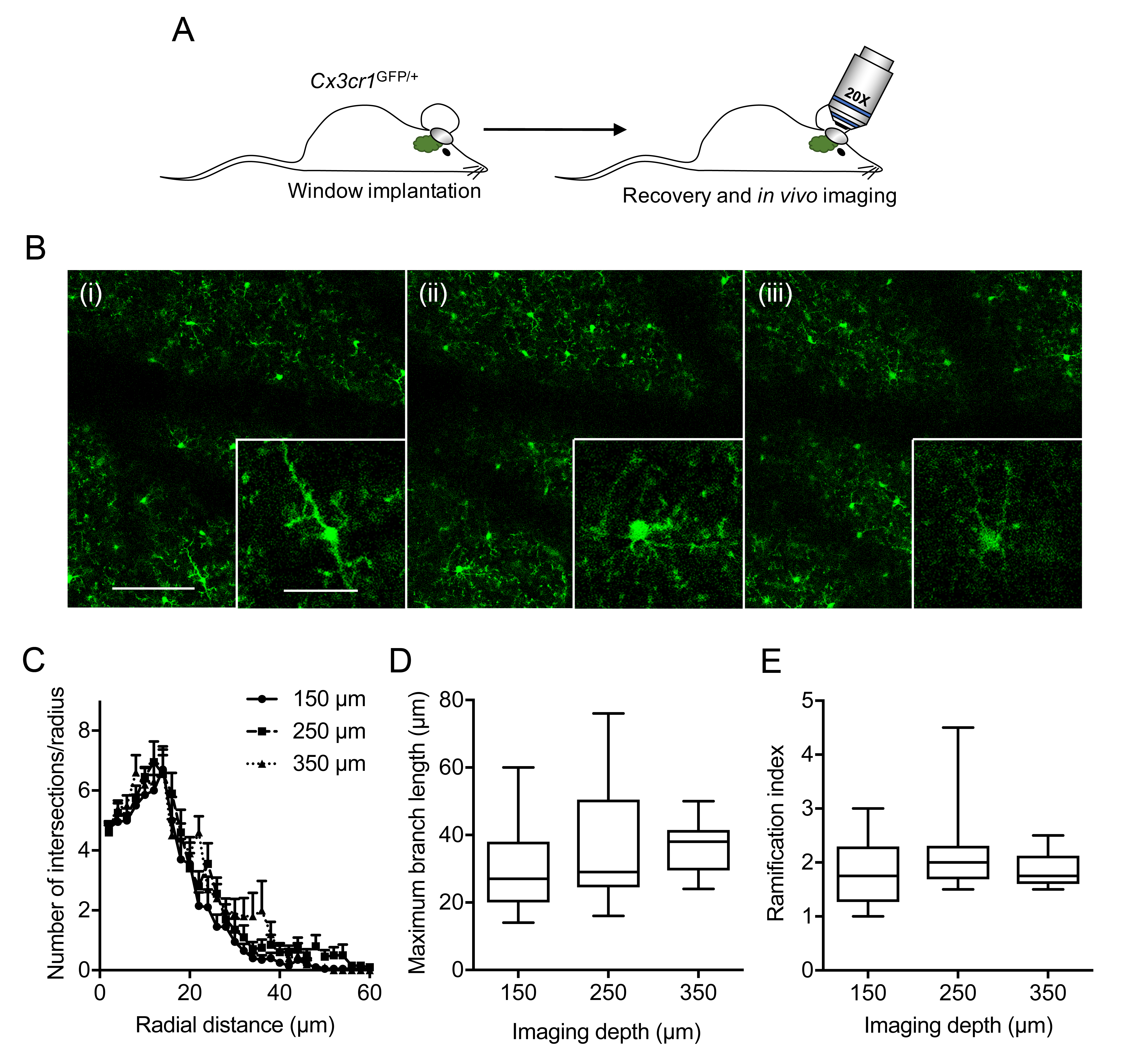

Supplement: Supplementary file 3 — Additional file 3: Supplementary Figure 3. GFP-expressing microglia are ramified in healthy mice in vivo. (A) Imaging window was implanted in Cx3cr1GFP/+ mice as described in Methods, mice allowed to recover, and microglia visualised under anaesthesia using adapted multiphoton microscope. (B) Representative images of microglia at various distances below the imaging window: 150 μm (i), 250 μm (ii), 350 μm (iii). Scale bar, 100 μm. Insets: 3X magnification; scale bar, 30 μm. (C) Sholl analysis plot of microglia imaged at the indicated depths below the imaging window (n = 10 cells/group). (D) Maximum branch length (μm) of microglia imaged at the indicated depths (n = 10 cells/group). (E) Schoenen ramification index of microglia at the indicated depths (n = 10 cells/group). Box plots show median, 25th and 75th percentile values; whiskers are minimum and maximum values. [file 12974_2020_1753_MOESM3_ESM.tif]

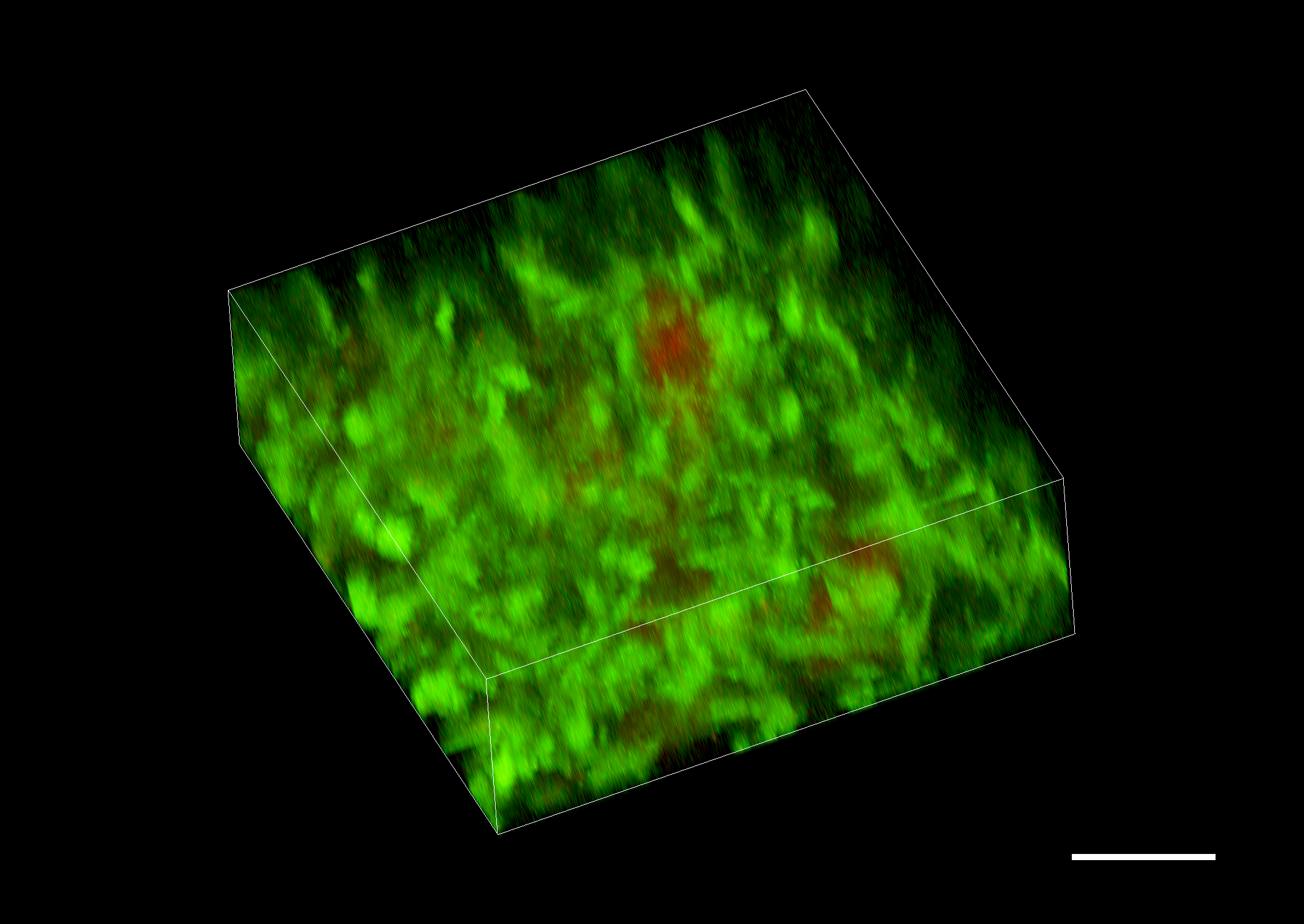

Supplement: Supplementary file 4 — Additional file 4: Supplementary Figure 4. 3D reconstruction of Z stack image of microglia and breast cancer cells. Microglia (green) were visualised under anaesthesia using adapted multiphoton microscope 7 days following implantation of DsRed-expressing tumour cells (red). Scale bar, 50 μm. [file 12974_2020_1753_MOESM4_ESM.tif]

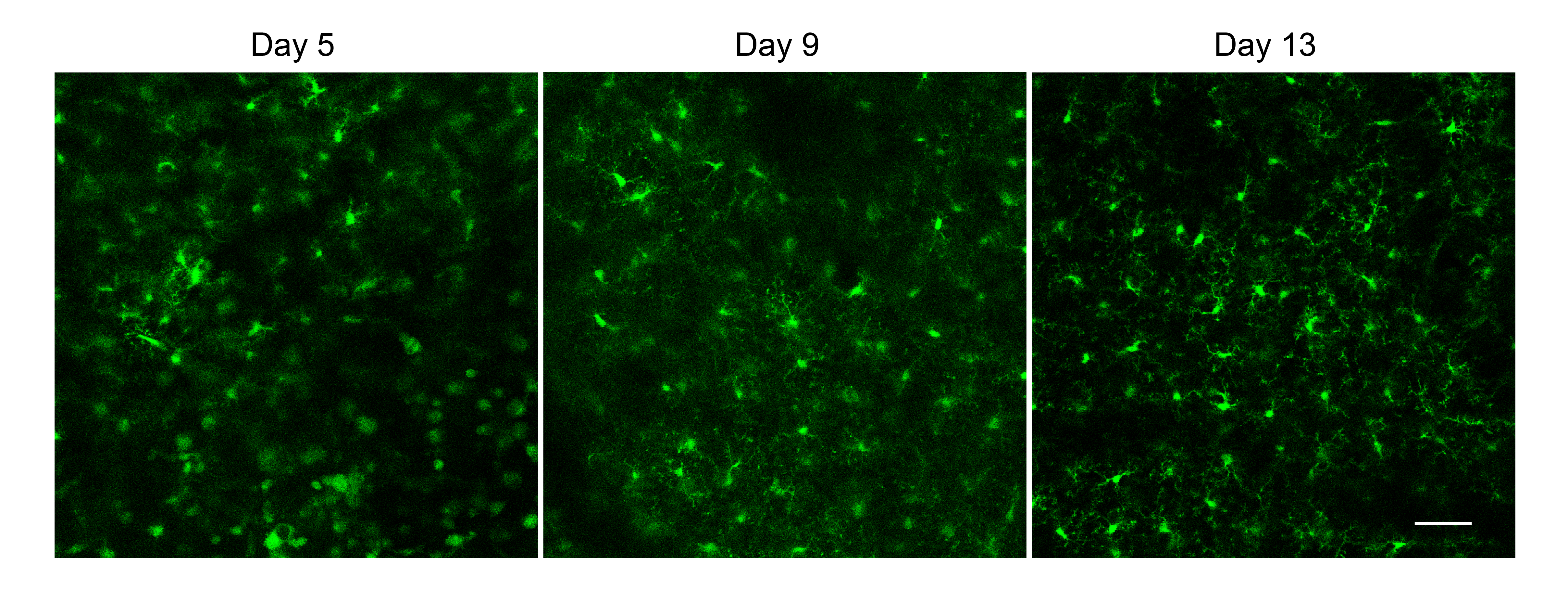

Supplement: Supplementary file 5 — Additional file 5: Supplementary Figure 5. Lack of effect of Matrigel on microglial morphology. Microglia (green) were visualised under anaesthesia using adapted multiphoton microscope at 5, 9, and 13 days following implantation of Matrigel alone without tumour cells. Scale bar, 50 µm. [file 12974_2020_1753_MOESM5_ESM.tif]

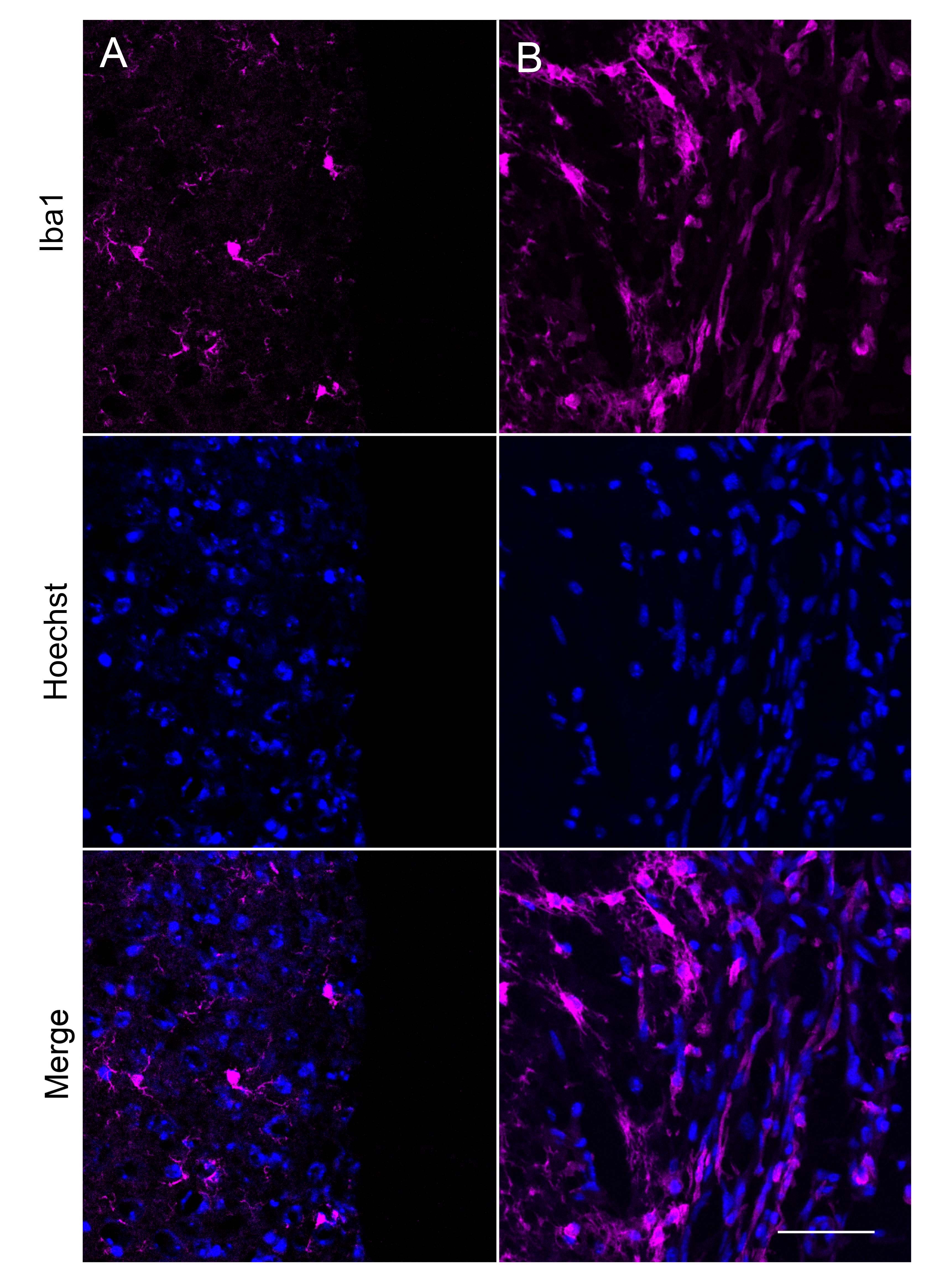

Supplement: Supplementary file 6 — Additional file 6: Supplementary Figure 6. Presence of Iba1-positive microglia at the lesion site. Confocal images at 63x of Iba1-positive microglia (magenta) and nuclei (blue) in coronal section obtained from a Cx3cr1GFP/+ mouse killed 6 days following implantation of tumour cells under the imaging window. (A) Control parenchyma away from lesion site. (B) Lesion site underneath location of imaging window. Scale bar, 50 µm. [file 12974_2020_1753_MOESM6_ESM.tiff]
