# Supplementary material for: Metastatic breast cancer cells induce altered microglial morphology and electrical excitability in vivo
Source: J Neuroinflammation. 2020 Mar 19;17:87. doi: 10.1186/s12974-020-01753-0 (PMC7081703; doi:10.1186/s12974-020-01753-0)
Supplement: Supplementary file 7 — Additional file 7 Table 1. Summary of Sholl analysis results from animals imaged in vivo and post mortem. [file 12974_2020_1753_MOESM7_ESM.docx]

**Table 1.** Summary of Sholl analysis results from animals imaged *in vivo* and *post mortem*.

| Parameter | Depth (µm) | *Post mortem* | *In vivo* | P value^1^ |
| --- | --- | --- | --- | --- |
| Maximum number of intersections/radius | 150 | 10.5 ± 0.8 | 8.5 ± 0.6 | 0.12 |
|  | 250 | 9.9 ± 1.0 | 9.5 ± 0.5 | 0.95 |
|  | 350 | 8.2 ± 0.5 | 8.7 ± 0.4 | 0.96 |
| Maximum branch length (µm) | 150 | 49.4 ± 5.2 | 28.8 ± 2.6 | < 0.001 |
|  | 250 | 39.0 ± 2.0 | 35.8 ± 3.6 | 0.88 |
|  | 350 | 39.2 ± 2.7 | 36.6 ± 2.6 | 0.96 |
| Ramification index | 150 | 2.2 ± 0.3 | 1.8 ± 0.1 | 0.35 |
|  | 250 | 2.1 ± 0.3 | 2.1 ± 0.1 | 0.99 |
|  | 350 | 1.8 ± 0.2 | 1.9 ± 0.1 | 0.96 |

^1^Two-way ANOVA (n ≥ 10 cells/group).
